# Nonalcoholic Fatty Liver Disease

**DOI:** 10.1155/2018/2097435

**Published:** 2018-07-31

**Authors:** Branka Filipović, Alastair Forbes, Bojan Tepeš, Dan L. Dumitraşcu

**Affiliations:** ^1^Clinical and Hospital Center “Bežanijska Kosa”, Autoput s/n, Medical Faculty, University of Belgrade, 4/2 Dr. Subotića Starijeg, 11000 Belgrade, Serbia; ^2^Norwich Medical School, Bob Champion Building, James Watson Road, University of East Anglia, Norwich Research Park, Norwich NR4 7UQ, UK; ^3^ABAKUS MEDICO d.o.o., Diagnostični Center Rogaška, Prvomajska 29, 3250 Rogaška Slatina, Slovenia; ^4^2nd Department of Internal Medicine, Iuliu Hatieganu University of Medicine and Pharmacy, Cluj-Napoca, Romania

Nonalcoholic fatty liver disease (NAFLD) is regarded as the most significant liver disease from the twenty-first century in the Western world. Although its development is surely driven by environmental factors, it is also regulated by genetic background. NAFLD ranges over a wide spectrum, extending from nonalcoholic fatty liver (NAFL) which is generally benign, through to nonalcoholic steatohepatitis (NASH) to liver cirrhosis, end-stage liver disease, and even hepatocellular carcinoma (HCC) despite the absence of significant alcohol consumption. The relationship of NAFLD with metabolic alterations such as type 2 diabetes is well described and related to insulin resistance, with NAFLD being recognized as the hepatic manifestation of metabolic syndrome. However, NAFLD may also coincide with endocrine diseases such as polycystic ovary syndrome, hypothyroidism, growth hormone deficiency, or hypercortisolism. It is therefore essential to remember, when discovering altered liver enzymes or hepatic steatosis on radiological exams, that endocrine diseases can cause NAFLD. In the latest years, obstructive sleep apnea syndrome (OSAS) has been associated NAFLD. Experimental evidence suggests that chronic intermittent hypoxia may per whole trigger liver injury, inflammation, and fibrogenesis, and, interestingly, OSAS is also believed to be one of the elements promoting the evolution of NAFLD from steatosis to nonalcoholic steatohepatitis (NASH) [1–4].

Z. Prokopowicz et al. are stressing the importance of the early diagnosis of NONAL in obese children and NAFLD predictive risk factors include increased waist circumference, elevated waist-hip ratio and waist-to-height ratio, and elevated total cholesterol, triglycerides, and fasting insulin as well as elevated glucose and insulin concentration in the OGTT and HOMA-IR index. NAFLD increases the risk of potential cardiovascular complications expressed by diagnosis of metabolic syndrome. The best independent predictive risk factor for diagnosing NAFLD in obese children is fasting insulin > 18.9 uIU/ml.

Shidfar et al. in their study aimed to examine the effect of virgin olive oil on alanine aminotransferase (ALT) and aspartate aminotransferase (AST) and the severity of steatosis in the NAFLD patients undergoing a weight-loss diet. Definitive conclusion is that the consumption of a low calorie diet enriched with olive oil, along with slight weight reduction, reinforces the desired effects of weight loss in improving the levels of the hepatic enzymes.

Y. Zhai et al. in their present paper investigated the association between NAFLD and sarcopenia in elderly patients and concluded that NAFLD is not independently associated with sarcopenia in older patients.

Miyata and Miyata studied the correlation between the period from the first to each examination date and the mean value of FIB4 index during the past year to each examination date was analyzed. In their conclusion, they claimed that correlation estimated was thought to be the time-dependent change of the mean FIB4 index during the past one year and in the present study the correlation was proved to be extremely strong. The time-dependent change of FIB4 index and its increase-decrease rate per year could be approximately speculated.

Our team (B. Filipović et al.) has been involved for a while in cognitive changes exploration among different gastroenterology patients. We hypothesized that NAFLD, as a condition, affects the brain tissue and, subsequently, the cognitive state. according to our results, patients with NAFLD had a greater risk to suffer from the cognitive impairment and depression: RR = 3.9; 95% CI 1.815–8.381; and RR = 1.65; 95% CI 1.16–2.36;  .Briefly, NAFLD significantly influenced the cognitive deficit and tissue volume reduction and patients suffering from NAFLD had about four times higher risk of having a cognitive impairment.

T. Milovanovic et al. investigated whether platelet count (PC), mean platelet volume (MPV), and platelet distribution width (PDW) can predict the presence of liver fibrosis in this group of patients. In their conclusion, patients with NAFLD have significantly higher values of PCT, PDW, and MPV when compared to the healthy controls. Further studies are needed to establish their potential use for prediction of the degree of liver steatosis and fibrosis in NAFLD patients.

V. Nguyen et al. have analyzed clinical, anthropometric, and biochemical changes at six months and after consecutive treatment with two and three serials of intragastric balloons (IGB). They concluded that IGB therapy is an effective, alternative nonsurgical means for weight loss in the management of obesity and NAFLD over the short term, with greatest outcomes observed after six months. Improvements in insulin resistance and hepatic transaminases correlated with weight change.

Apparently, according to the papers published in this special issue, NAFLD is a serious problem, which each author from their own aspect tried to clarify. Regarding the fact that NAFLD is rarely isolated and that it is correlated with obesity, diabetes type 2, polycystic ovarian syndrome, obstructive sleep apnea, and some cognitive deficits, its pathophysiology and clinical development require more investigations. Suggestions for the treatment by the implantation of the intragastric balloon must be considered as one of the treating solutions in the future.

*Branka Filipović* *Alastair Forbes* *Bojan Tepeš* *Dan L. Dumitrascu*
